# The usefulness of total body protein mass models for adolescent athletes

**DOI:** 10.3389/fnut.2024.1439208

**Published:** 2024-07-08

**Authors:** Analiza M. Silva, Francesco Campa, Luís B. Sardinha

**Affiliations:** ^1^Exercise and Health Laboratory, CIPER, Faculdade Motricidade Humana, Universidade de Lisboa, Lisbon, Portugal; ^2^Department of Biomedical Sciences, University of Padua, Padua, Italy

**Keywords:** body composition, adolescent athletes, total body protein, DXA, multicomponent models

## Abstract

The present study aimed to assess the utility of a less laborious technique for estimating total body protein (TBPro) in young athletes, using a multicomponent model as the criterion method. A total of 88 (49 boys and 39 girls) adolescent athletes (age: 15.2 ± 1.5 years; body mass index: 21.2 ± 2.7 kg/m^2^) participated. A 6-compartment model was used as the reference method (TBPro_Reference_) involving air displacement plethysmography for body volume, dual-energy X-ray absorptiometry (DXA) for bone mineral content, and deuterium dilution for total body water (TBW). Alternatively, DXA TBPro models were used as TBPro = lean-soft mass (LSM) − HF_FFM_ × fat-free mass (FFM) − Ms. − G, where LSM and FFM were assessed using DXA, HF_FFM_ is the hydration fraction of the FFM using measured TBW or assumed TBW (adult fraction of 0.732; Lohman’s constants or mean observed HF_FFM_), Ms. is soft tissue minerals (Ms = 0.0129 × HF_FFM_ × FFM), and G is glycogen calculated as 0.044 × (LSM − HF_FFM_ × FFM − Ms). The maturation level was determined by self-assessment. TBPro obtained from DXA using the assumed HF_FFM_ explained 73% to 77% of the variance compared to TBPro_Reference._ Meanwhile, using the mean values of measured HF_FFM_, the DXA model explained 53 and 36% for boys and girls, respectively. Larger bias (8.6% for boys and 25.8% for girls) and limits of agreement were found for the DXA model using measured HF_FFM_ (boys for 66.9% and girls for 70%) compared to an assumed HF_FFM_ (bias ranged from 1.5% to 22.5% and limits of agreement ranged from 31.3% to 35.3%). Less complex and demanding TBPro DXA models with the assumed HF_FFM_ are valid alternatives for assessing this relevant FFM component in groups of adolescent athletes but are less accurate for individual results. Though future studies should be conducted to test the usefulness of these models in longitudinal and experimental designs, their potential to provide an estimation of protein mass after exercise and diet interventions in young athletes is anticipated.

## 1 Introduction

Protein is the second major component of the fat-free mass (FFM) (1), i.e., on a molecular level of body composition analysis, and plays a central role in many biochemical and physiological processes (2). Total body protein (TBPro) increases during growth and development, as well as in response to exercise programs, specifically strength and resistance training, coupled with adequate levels of dietary protein intake (3, 4).

The evaluation of TBPro in athletes is particularly relevant, namely in tracking the overall nutritional status, but also in calibrating and adjusting the diet to fit their respective whole-body protein needs specific to their respective sport (3, 5). Typically, TBPro assessment *in vivo* is obtained with complicated and expensive techniques using *in vivo* neutron activation analysis (IVNA). However, the application of IVNA is limited because it is cost-prohibitive and involves radiation exposure (6).

Traditional methods to assess molecular components are based on a two-component (2C) approach (7), either using hydrostatic weighing or air displacement plethysmography, assuming constant densities for FFM density and hydration based on human cadaver analysis (8) that compromise a valid body composition assessment in the pediatric population (9). Multicomponent models such as the four-compartment molecular model (4C model: fat, water, mineral, and protein/residual) have been used to validate 2C models in pediatric groups (9, 10), providing a protein mass component (or a residual mass) that can be further adjusted for non-protein components typically found in residual mass (11). Therefore, a six-compartment model [6C model: fat, water, protein, bone mineral, soft tissue mineral, and glycogen (G)] can also be employed to accurately assess TBPro by including measurements of body mass, body volume (BV), bone mineral content (BMC), total body water (TBW), and assumptions for soft tissue minerals (Ms) and G (1, 11, 12). Additionally, a model based on whole-body dual-energy X-ray absorptiometry (DXA) (13) can be used to estimate TBPro. The DXA systems allow measurements of BMC and lean soft mass (LSM), which compose the basis of an assessment of TBPro, and are both less expensive and generally more accessible than IVNA. Theoretically, LSM is considered to consist of TBW, Ms., TBPro, and G because other components are quantitatively less significant (14). Hence, TBPro may be estimated by subtracting TBW, Ms., and G from LSM. Dilution techniques are the gold standard for estimating TBW *in vivo* (15). Alternatively, TBW may be predicted by assuming that FFM, equivalent to the sum of DXA measurements of BMC and LSM, has a constant hydration fraction (approximately 73.2% in adults) (16), which ranges from 69% to 78% in children based on gender and age (17).

Heymsfield et al. (11) presented the comparability of a 6C model, including estimates of Ms. and G, against the reference approach, IVNA, in healthy adults. The authors observed that the TBPro estimation from the 6C model was minimally larger than the IVNA TBPro, though highly correlated and with no significant bias (11). Previous studies using 4C models were conducted on young athletes exploring TBPro without adjusting for Ms. and G and observed an 8% and 13% increase in this component over one season (18). Recognizing the relevance of assessing and tracking changes in TBPro in athletes, simple alternatives, such as the TBPro-DXA-based models, should be explored. Though the accuracy of TBPro estimates by DXA-based models has been studied in children and adults using a 4C model as the reference method for TBPro (19), its accuracy for measuring TBPro is unknown in a sample of adolescent athletes. Therefore, the purpose of the current study was to assess the usefulness of DXA models to estimate TBPro with either measured or assumed TBW constants in adolescent athletes using a 6C model as the reference method.

## 2 Materials and methods

### 2.1 Subjects

A total of 88 Portuguese adolescent athletes, 39 girls (age 15.1 ± 1.8 years) and 49 boys (age 15.4 ± 1.1 years), volunteered to participate in this study. Subjects were recruited from five sports clubs in Lisbon, Portugal, and were involved in several professional sports (swimming, basketball, rugby, gymnastics, and judo). The parents/guardians of each participant and the athletes involved in the investigation were given written and verbal instructions regarding the research design and all the procedures required for the study. After informing each participant and their parent/guardian about the investigation protocol, possible risks, and benefits of participating in the study, a signed written consent form was obtained. All procedures and consent forms were approved by the Ethical Committee of the Faculty of Human Kinetics, University of Lisbon.

The inclusion criteria for the athletes were: (i) current participation in competitive sports at national and international levels; (ii) at least 2 h per week of training; and (iii) 3 years or more of training in their respective sport. The exclusion criteria were: (i) athletes taking medication for illness or injuries, (ii) any kind of supplements; (iii) female athletes who reported to be amenorrhoeic; and (iv) athletes who were in the process of gaining or losing weight. Information about each subject’s sports participation, medical history, medication, and diseases was collected through a questionnaire.

### 2.2 Maturation

The subjects were grouped according to pubertal status, determined by self-assessment using the Tanner stage (20) adapted by Ross and Marfell-Jones (21). A self-evaluation method with figures was used to identify the degree of development of the genital organs, breasts, and pubic hair.

### 2.3 Procedures

All measurements were performed on the same day, during one visit to the study laboratory, over a 4-h period. The measurements were performed by the same technician. Participants were asked to fast from the previous evening and to avoid moderate or vigorous exercise training intensity in the previous 24 h. After an overnight fast of 8–10 h, the subjects came to the laboratory, where all measurements were carried out. The measurements started with the assessment of body mass and height. Next, the participant received a dose of deuterium oxide for TBW assessment, followed by a whole-body DXA scan and then BV assessment.

### 2.4 Body composition measurements

#### 2.4.1 Body weight and height

Body weight was measured twice using an electronic scale (SECA model 770; Hamburg, Germany) to the nearest 0.1 kg, and the average was recorded as their weight. Stature was also measured twice, without shoes, to the nearest 0.1 cm, and the average was recorded as their height.

#### 2.4.2 Body volume

BV was assessed using an air displacement plethysmograph (BOD POD®, COSMED, Italy). Each subject wore a swimming suit, and their body mass was measured to the nearest 100 g using an electronic scale connected to a plethysmograph computer. BV was computed based on the initial BV corrected for thoracic gas volume and a surface area artifact was computed automatically (22). Measured thoracic gas volume was measured in all subjects.

All measurements were conducted using the BOD POD® software version 1.68. Based on the test–retest performed on 10 athletes, the CV value in our laboratory for measuring BV is 0.4% (23).

#### 2.4.3 Total body water

TBW was assessed using the deuterium dilution technique with a stable Hydra gas isotope ratio mass spectrometer (PDZ, Europa Scientific, United Kingdom). After completing a 12-h fast, an initial urine sample was collected, at which time the subject was immediately administered deuterium oxide (^2^H_2_O) at a dose of 0.1 g/kg of body weight, diluted in 30 mL of water. After a 4-h equilibration period, another urine sample was collected. Abundances of ^2^H_2_O in dilutions of the isotope doses were then analyzed. The urine and diluted dose samples were prepared for ^1^H/^2^H analysis using the equilibration technique of Prosser and Scrimgeour (24). After the tubes were filled with zinc, they were equilibrated at 20°C ± 1°C overnight for 3 days. The tubes were then introduced sequentially into a helium flow that was dried by magnesium perchlorate and then analyzed using a Hydra gas isotope ratio mass spectrometer set to detect ^1^H/^2^H. The enrichments of equilibrated local water standards were calibrated against Standard Mean Ocean Water (SMOW). Based on delta SMOW, TBW was estimated, including a 4% correction due to the dilution of deuterium into non-aqueous space (15). Our laboratory’s CV for measuring TBW, based on a test–retest involving 10 subjects, is 0.3% (23).

#### 2.4.4 TBPro from the multicomponent molecular model

A six-compartment model using assumptions for Ms (Ms = 0.0129 × TBW) (12) and G (G = 0.044 × TBPro) was used as the criterion method to estimate TBPro (1). TBPro was estimated from the reference method as body mass minus fat mass (FM), TBW, Mo, and the assumed calculations for Ms. and G, as described:


(1) $$\begin{array}{l} \text{TBPro}=2.922\times BM-0.301\times TBW-2.039\times Mo\\ -2.632\times BV \end{array}$$

where BV is body volume (L), TBW is total body water (kg), Mo is total body bone mineral (kg), Ms. is soft tissue mineral (kg), and BM is body mass (kg).

#### 2.4.5 TBPro estimated from DXA

The BMC, LSM, and FM were estimated using a DXA (Hologic Explorer-W, fan beam densitometer, software QDR for Windows version 12.4, Waltham, MA, United States). A whole-body scan was performed and the attenuation of X-rays pulsed between 70 and 140 kV synchronously with the line frequency for each pixel. Following the protocol for DXA described by the manufacturer, a step phantom with six fields of acrylic and aluminum of varying thickness and known absorptive properties was scanned to serve as an external standard for the analysis of different tissue components. The same laboratory technician positioned the subjects, performed the scans, and executed the analysis according to the operator’s manual using the standard analysis protocol. Considering that BMC represents ashed bone, BMC was converted to Mo by multiplying it by 1.0436 (25). TBPro can be then estimated by:


(2) $${\text{TBPro}}_{DXA}=LSM-{HF}_{FFM}\times FFM-Ms.-G$$

where LSM was determined by DXA, HF_FFM_ x FFM is equivalent to either measured TBW or using an assumed TBW fraction of DXA FFM [HF_FFM_ = 0.732 (AdultHF_FFM_) (26); HF_FFM_ from Lohman’s constants (LohmanHF_FFM_) (17); and mean measured HF_FFM_ for boys and girls (meanObsHF_FFM_)], Ms. was calculated as 0.0129 × HF_FFM_ × FFM, and G was assessed as (LSM − HF_FFM_ × FFM − Ms) × 0.044. The CV for BMC in our laboratory based on a test–retest involving 10 subjects was 1.3% (23).

#### 2.4.6 Propagation of measurement error

In this study, the propagation of measurement error associated with TBPro determination from the multicomponent model can be estimated by using the measurement precision of each method involved in the determination of BV, TBW, Mo, and body mass and assuming an average body composition of the whole sample (BV, TBW, Mo, and body mass), as stated throughout this section. Accordingly,


TBPro=2.922×BM−0.301×TBW−2.039×Mo−2.632×BV

$$\begin{array}{l} \text{TBPro}\;\nu^{2}={\left(2.922\times 62.0\times 0.0007\right)}^{2}+{\left(0.301\times 37.5\times 0.003\right)}^{2}\\ +{\left(2.039\times 2.73\times 0.013\right)}^{2}+{\left(2.632\times 58.3\times 0.004\right)}^{2}\\ =0.399\;\left(\nu =0.632\;\text{kg}\right) \end{array}$$

The test–retest reliability data collected in the present study thus yields a value of ~0.6 kg.

### 2.5 Statistical analysis

Paired t-tests were used to compare TBPro from the DXA models with the 6C model, for boys and girls. A simple linear regression analysis was performed when comparing TBPro estimates by the DXA models, as the independent variables, with the reference method, as the dependent variable. A multiple regression analysis was performed to test the influence of maturation level alone and in interaction with TBPro obtained by each DXA model, separately. For each TBPro-DXA model, if the maturation level and the interaction term between this factor and TBPro from the DXA models were non-significant, a simple linear regression analysis would be performed to explain TBPro from the reference method without dividing the sample by maturation level. The standard error of estimation (SEE) and the coefficient of correlation (r) were analyzed. The SEE was used as a measure of validation to assess the lack of association between the two methods (TBPro from the reference method vs. TBPro from each DXA model). Agreement between methods was assessed, including the 95% limits of agreement (27). Statistical significance was set at a *p*-value of <0.05.

## 3 Results

The means and standard deviation values of body composition results are displayed in Table 1.

**Table 1:** Subject characteristics.

	Girls (*n* = 39)	Boys (*n* = 49)	Total (*n* = 88)
	Mean ± SD		Mean ± SD
Age (years)	15.1 ± 1.8	15.4 ± 1.1	15.2 ± 1.5
Weight (kg)	56.1^2^ ± 13.5	66.6 ± 11.0	62.0 ± 13.1
Stature (m)	1.65^2^ ± 0.13	1.74 ± 0.08	1.70 ± 0.11
BMI (kg/m^2^)	20.4^2^ ± 2.5	21.9 ± 2.6	21.2 ± 2.7
BV (L)	53.3^2^ ± 13.4	62.3 ± 10.6	58.3 ± 12.6
%FM_Reference_	20.0^2^ ± 6.8	12.4 ± 5.5	15.8 ± 7.2
%FM_DXA_	23.9^1,2^ ± 6.8	13.4^1^ ± 5.3	18.0^1^ ± 8.0
FFM_Reference_	44.2^2^ ± 8.2	58.2 ± 9.1	52.0 ± 11.1
FFM_DXA_ (kg)	41.6^1,2^ ± 7.8	57.1^1^ ± 8.8	50.2^1^ ± 11.4
LSM_DXA_ (kg)	39.3^2^ ± 7.2	54.2 ± 8.2	47.6 ± 10.8
Mo (kg)	2.41^2^± 0.67	2.99 ± 0.61	2.73 ± 0.70
TBW (kg)	31.6^2^ ± 6.1	42.2 ± 6.7	37.5 ± 8.3
TBW/FFM_6C_	0.715^2^ ± 0.018	0.725 ± 0.019	0.720 ± 0.019
LohmanHF_FFM_	0.752^2,3^ ± 0.005	0.743^3^ ± 0.003	0.747^3^ ± 0.006
TBPro_Reference_ (kg)	9.3^2^ ± 1.6	12.0 ± 2.0	10.8 ± 2.2
TBPro-DXA_TBW_ (kg)	6.9^2,4^ ± 2.0	11.0^4^ ± 2.7	9.2^4^ ± 3.1
TBPro-DXAadultHF_FFM_ (kg)	8.0^2,4^ ± 1.4	11.3^4^ ± 1.7	9.9^4^ ± 2.2
TBPro-DXALohmanHF_FFM_ (kg)	7.2^2,4^ ± 1.5	10.8^4^ ± 1.6	9.2^4^ ± 2.4
TBPro-DXAmeanObsHF_FFM_ (kg)	8.8^2,4^ ± 1.5	11.8 ± 1.7	10.5^4^ ± 2.2

BV, body volume; FM, fat mass; %FM_Reference_, percent fat mass from the reference six-compartment model; FFM_Reference_, fat-free mass from the reference six-compartment model; FFM_DXA_, fat-free mass from DXA; LSM_DXA_, lean-soft mass from DXA; TBW, total body water; Mo, bone mineralTBW/FFM_6C_, hydration fraction of the reference fat-free mass; LohmanHF_FFM_, Lohman’s FFM hydration; TBPro, total body protein; TBPro_Reference_ reference total body protein; TBPro-DXA_TBW_, total body protein using measured total body water; TBPro-DXAadultHF_FFM_, total body protein using the adult assumed fat-free mass hydration (0.732); TBPro-DXALohmanHF_FFM_, total body protein using Lohman’s constants for fat-free mass hydration; TBPro-DXAmeanObsHF_FFM_, total body protein using mean observed hydration fraction of the reference fat-free mass. ^1^Significantly different from the %FM_Reference_ *p* < 0.05. ^2^Significantly different between boys and girls, *p* < 0.05. ^3^Significantly different from TBW/FFM_Reference_, *p* < 0.05. ^4^Significantly different from TBPro_Reference_, *p* < 0.05.

The maturation level (as a main effect) and the interaction term between this factor and TBPro from each DXA model were non-significant for girls (*p* > 0.05) and boys (*p* > 0.05) in explaining reference TBPro, enabling the use of the whole sample by gender, regardless of the maturation status. For both genders, the prediction of TBPro from using DXA-based models incorporating measured TBW or assuming adult FFM hydration (0.732), the mean measured value of FFM hydration (0.715 ± 0.018) obtained in girls, and Lohman’s FFM hydration constants significantly underestimated TBPro from the reference method (*p* < 0.05), except when the mean measured value of FFM hydration (0.725 ± 0.019) obtained in boys was used (*p* > 0.05).

DXA models with an assumed hydration fraction (AdultHF_FFM_, MeanObsHF_FFM_, and LohmanHF_FFM_) explained 77% and 74%, 77% and 73%, and 77% and 74% of the values obtained from the criterion TBPro variance for boys and girls, respectively. However, when measured TBW was incorporated into the DXA model, an explained variance of 53 and 36% was observed, as indicated in Table 2. Furthermore, TBPro estimations from the DXA-based model with measured TBW revealed a higher standard error of measurement (boys: 1.37 kg; girls: 1.26 kg) in predicting TBPro_Reference_ compared to the other DXA-based models using an assumed FFM hydration, ranging from 0.80 to 0.96 kg.

**Table 2:** Regression parameters for TBPro estimation from DXA-based models against the reference method.

	Slope	Intercept	r	SEE
**TBPro-DXA**_**TBW**_				
Girls (*n* = 39)	0.47^2^	6.09^1^	0.60	1.26
Boys (*n* = 49)	0.54^2^	6.07^1^	0.73	1.37
**TBPro-DXAadultHF**_**FFM**_				
Girls (*n* = 39)	0.98	1.46	0.86	0.80
Boys (*n* = 49)	1.04	0.14	0.88	0.96
**TBPro-DXAmeanObsHF**_**FFM**_				
Girls (*n* = 39)	0.89	1.53	0.86	0.80
Boys (*n* = 49)	1.03	0.14	0.88	0.95
**TBPro-DXALohmanHF**_**FFM**_				
Girls (*n* = 39)	0.91	2.78^1^	0.85	0.82
Boys (*n* = 49)	1.05	0.62	0.88	0.96

r, coefficient of correlation; SEE, standard error of estimate; TBPro_Reference_ reference total body protein; TBPro-DXA_TBW_, total body protein using measured total body water; TBPro-DXAadultHF_FFM_, total body protein using the adult assumed fat-free mass hydration (0.732); TBPro-DXALohmanHF_FFM_, total body protein using Lohman’s constants for fat-free mass hydration; TBPro-DXAmeanObsHF_FFM_, total body protein using mean observed hydration fraction of the reference fat-free mass. ^1^Significantly different from 0, *p* < 0.05. ^2^Significantly different from 1, *p* < 0.05.

Slope and intercept differed from the line of identity (*p* < 0.05) for DXA with TBW measures, whereas the DXA models assuming FFM hydration did not show significant differences (*p* > 0.05) for both genders.

At an individual level, larger limits of agreement were found for the DXA model with measured TBW (boys: −4.6 to 2.7 kg; girls: −5.7 to 0.9 kg) compared to the DXA model using the adult FFM hydration value (boys: −2.5 to 1.3 kg; girls: −2.9 to 0.3 kg), the mean observed FFM hydration in our sample (boys: −2.1 to 1.7 kg; girls: −2.2 to 1.0 kg), or when Lohman’s constants were applied (boys: −3.1 to 0.8 kg; girls: −3.7 to −0.5 kg).

**Figure 1:**
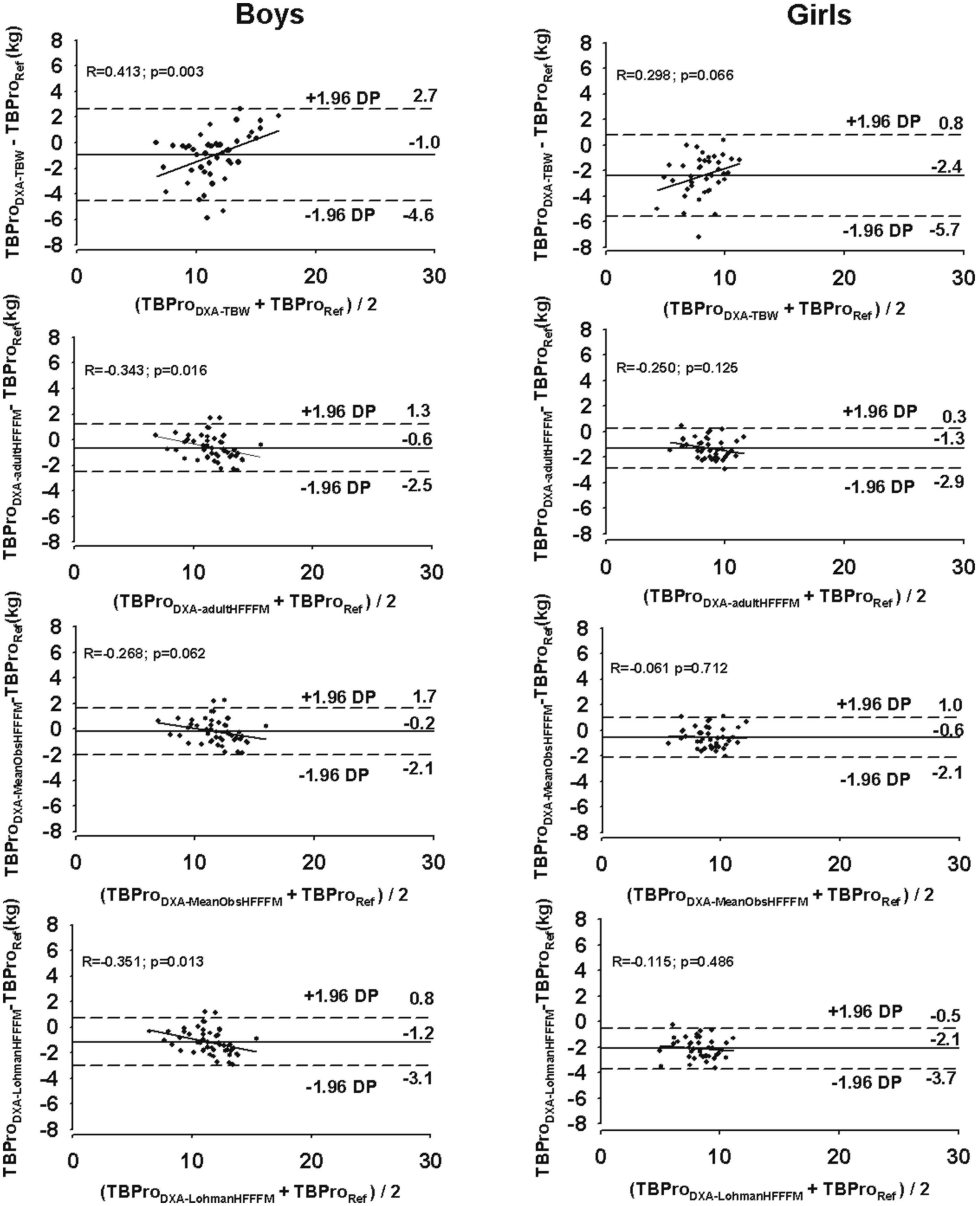
Agreement between the reference method (TBPro_Reference_, total body protein using the six-compartment model) and the DXA-based TBPro models (TBPro_DXATBW_, total body protein using measured total body water; TBPro_DXAadultHFFFM_, total body protein using the adult assumed fat-free mass hydration (0.732); TBPro_DXALohmanHFFFM_, total body protein using Lohman’s constants for fat-free mass hydration; TBPro_DXAmeanObsHFFFM_ total body protein using mean observed hydration fraction of the reference fat-free mass) for boys (right panels) and girls (left panels). The solid line represents the mean differences between the reference method and each DXA model. The dashed lines represent 95% limits of agreement (±1.96 SD). The trend line represents the association between the differences between the methods and the mean of both methods.

For girls, no trend was found between the differences between the methods and the mean of both methods (*p* > 0.05), while for boys, a significant trend was observed for all TBPro-DXA models (*p* < 0.05), except when TBPro was estimated using the mean FFM hydration value found in our male adolescent athletes (DXA-MeanObsHF_FFM_), as illustrated in Figure 1.

## 4 Discussion

The main finding about the usefulness of DXA-based models for TBPro mass estimation reveals that when assumed FFM hydration values are incorporated, the accuracy in assessing this FFM component is improved compared to measured FFM hydration in adolescent athletes, though a systematic underestimation is observed, except in boys using the mean observed FFM hydration.

A 6C model was chosen as an appropriate reference method against which to evaluate the DXA model of TBPro. This multicomponent model involves the inclusion of measured values for TBW and Mo, which serves to eliminate uncertainties associated with the assumptions necessary for less complex models (e.g., two-component models) of body composition assessment (28, 29). The 6C model reduced the systematic presence of other body components not taken into account by the 4C model, such as G and non-osseous minerals. Wang et al. (1) proposed accounting for G by assuming that this component constitutes approximately 44 g/kg of TBPro (30). For the Ms. component, a stable relationship was found between Ms. and TBW (12). We anticipate that this is a major strength of this study since fewer assumptions were made. It is generally accepted that DXA-derived FM and LSM are less valid compared to BMC measurements. This is partly because DXA estimates of FM and LSM are assumed to be in the same proportions in pixels containing bone as they are in pixels with no bone and partly due to the confounding effects of tissue thickness (31–33). Similarly, assessments of TBPro by the DXA method are less valid than those for the reference 4C model when measured TBW is taken into account, but slightly more accurate when an assumed constant for FFM hydration is applied (13), by limiting the biological variability in TBW measurements. These authors further indicated that TBPro improves significantly when estimated TBW, rather than measured TBW, is integrated into the DXA model (13). Our findings extend these results, using a 6C model with the assumed stable relationship for G and Ms. It is important to note that our DXA instrument underestimated FFM in female athletes by ~3 kg and more than 1 kg in male athletes.

The estimates obtained using measured TBW in our adolescents are further confounded by the significant relationship between measurement differences and the size of the estimation. The improvement in predicting TBPro using an assumed FFM hydration is possibly explained by the larger biological variability when the individual-measured TBW is incorporated instead of an assumed constant. Nevertheless, even when using an assumed FFM hydration, it should be noted that the difference between TBPro-DXA-based models and the criterion would increase (i.e., increasing the error for calculating TBPro) as the individual FFM hydration deviates further from the assumed constancy. Indeed, as the TBW to FFM ratio increases (17), which is the case when Lohman’s age-adjusted constants were used, the larger the absolute differences between DXA-based models and the reference method for TBProt assessment. Although the present study used a sample of adolescent athletes, with an expected higher contribution of TBW within the FFM, Lohman’s age-adjusted hydration constants were not useful given that the FFM hydration fraction observed in girls was 71.5% and in boys 72.5% (values even below the adult FFM hydration of 73.2%). Therefore, TBPro was markedly underestimated when these constants were used.

In the present study, we have assumed that the 6C models offer the most accurate estimates of TBPro. Wang et al. (25) provide further details on the assumptions for multicomponent model development. However, it is important to note that the gold standard method for TBPro assessment is obtained from total body nitrogen using IVNA (11). Though the current findings may be treated with caution, IVNA systems are costly to construct and expose subjects to ionizing radiation (~0.26 mSv).

Another methodological limitation is the use of a stable relationship between TBPro and G. We assumed that 44 g/kg TBPro is presented in our adolescent sample. Nevertheless, the FFM proportion of the main components observed in our young athletes differed from the common pediatric population (lower FFM hydration), as factors such as nutritional status and exercise may affect this relation (4, 34).

Given that body composition differs between DXA manufacturers and model equipment (35, 36), these results may not be generalized for other DXA manufacturers and models.

Hydration status was not determined, for instance, by using urine-specific gravity, which means that it was not possible to ensure whether athletes were considered euhydrated. Nonetheless, a recent study aimed at comparing body water compartments and hydration status of athletes with different regular amounts of water intake (low vs. high water drinkers with higher and lower urine specific gravity values) found no differences in TBW and FFM hydration between groups for both sexes (37).

Another limitation that should be addressed is the potential impact of the athletic background, specifically, the competitive level, years of experience, volume of training, and even player position, on the between-differences in TBPro assessment.

Finally, while the Tanner stages provide a useful framework for assessing pubertal development, self-assessment is inferior to clinical assessment (such as wrist radiography) due to subjectivity, cultural and individual variability, ethical concerns, and the narrow focus on physical characteristics (38), adding potential inaccuracies in pubertal status determination in this study.

## 5 Conclusion

Overall, the incorporation of assumed FFM hydration values in the DXA-based models appears to improve its accuracy compared to the inclusion of measured TBW fraction by limiting the biological variability of TBW and its impact on the explained variance of reference TBPro values. The DXA models using assumed FFM hydration have the potential to improve research and clinical practice in group studies of adolescent athletes but are less accurate for individual values. Measurements of TBPro *in vivo* can provide important information regarding healthy growth and development but also track the effects of exercise and diet interventions toward FFM gains in the pediatric athletic population. Nevertheless, more research is required to assess the accuracy of these models in tracking TBPro changes in young athletes using longitudinal and experimental designs.

## Data availability statement

The raw data supporting the conclusions of this article will be made available by the authors, without undue reservation.

## Ethics statement

The studies involving humans were approved by Ethical Committee of the Faculty of Human Kinetics, University of Lisbon. The studies were conducted in accordance with the local legislation and institutional requirements. Written informed consent for participation in this study was provided by the participants’ legal guardians/next of kin.

## Author contributions

AS: Conceptualization, Formal analysis, Investigation, Methodology, Project administration, Writing – original draft, Writing – review & editing. FC: Conceptualization, Methodology, Writing – original draft. LS: Conceptualization, Supervision, Writing – review & editing.

## References

[1] Wang Z Heshka S Wang J Heymsfield SB. Total body protein mass: validation of total body potassium prediction model in children and adolescents. J Nutr. (2006) 136:1032–6. doi: 10.1093/jn/136.4.1032, PMID: 16549470

[2] Silva AM. Structural and functional body components in athletic health and performance phenotypes. Eur J Clin Nutr. (2019) 73:215–24. doi: 10.1038/s41430-018-0321-9, PMID: 30287933

[3] Hartman JW Moore DR Phillips SM. Resistance training reduces whole-body protein turnover and improves net protein retention in untrained young males. Appl Nutr Metab. (2006) 31:557–64. doi: 10.1139/h06-031, PMID: 17111010

[4] Jäger R Kerksick CM Campbell BI Cribb PJ Wells SD Skwiat TM. International Society of Sports Nutrition Position Stand: protein and exercise. J Int Soc Sports Nutr. (2017) 14:20. doi: 10.1186/s12970-017-0177-8, PMID: 28642676 PMC5477153

[5] Kerksick CM Arent S Schoenfeld BJ Stout JR Campbell B Wilborn CD. International society of sports nutrition position stand: nutrient timing. J Int Soc Sports Nutr. (2017) 14:33. doi: 10.1186/s12970-017-0189-4, PMID: 28919842 PMC5596471

[6] Cohn SH Parr RM. Nuclear-based techniques for the in vivo study of human body composition. Report of an advisory Group of the International Atomic Energy Agency. *Clin. Phys. Physiol. Meas. An off. J. Hosp. Phys. Assoc. Dtsch. Gesellschaft fur Medizinische Phys*. Eur Fed Organ Med Phys. (1985) 6:275–301. doi: 10.1088/0143-0815/6/4/001 3907934

[7] Siri WE. Body composition from fluid spaces and density: analysis of methods. 1961. Nutrition. (1993) 9:480–91. 8286893

[8] Brozek J Grande F Anderson JT Keys A. DENSITOMETRIC analysis of body composition: revision of some quantitative assumptions. Ann N Y Acad Sci. (1963) 110:113–40. doi: 10.1111/j.1749-6632.1963.tb17079.x, PMID: 14062375

[9] Fields DA Goran MI McCrory MA. Body-composition assessment via air-displacement plethysmography in adults and children: a review. Am J Clin Nutr. (2002) 75:453–67. doi: 10.1093/ajcn/75.3.453, PMID: 11864850

[10] Wells JC Fuller NJ Dewit O Fewtrell MS Elia M Cole TJ. Four-component model of body composition in children: density and hydration of fat-free mass and comparison with simpler models. Am J Clin Nutr. (1999) 69:904–12. doi: 10.1093/ajcn/69.5.904, PMID: 10232629

[11] Heymsfield SB Ebbeling CB Zheng J Pietrobelli A Strauss BJ Silva AM. Multi-component molecular-level body composition reference methods: evolving concepts and future directions. Obes Rev. (2015) 16:282–94. doi: 10.1111/obr.12261, PMID: 25645009 PMC4464774

[12] Wang Z Pi-Sunyer FX Kotler DP Wielopolski L Withers RT Pierson RNJ. Multicomponent methods: evaluation of new and traditional soft tissue mineral models by in vivo neutron activation analysis. Am J Clin Nutr. (2002) 76:968–74. doi: 10.1093/ajcn/76.5.968, PMID: 12399267

[13] Fuller NJ Wells JC Elia M. Evaluation of a model for total body protein mass based on dual-energy X-ray absorptiometry: comparison with a reference four-component model. Br J Nutr. (2001) 86:45–52. doi: 10.1079/bjn2001387, PMID: 11432764

[14] Pietrobelli A Formica C Wang Z Heymsfield SB. Dual-energy X-ray absorptiometry body composition model: review of physical concepts. Am J Phys. (1996) 271:E941–51. doi: 10.1152/ajpendo.1996.271.6.E941 8997211

[15] Shoeller D. Hydrometry. In: SB Heymsfield TG Lohman Z Wang S Going, editors. The human body composition. Champaign, IL: Human Kinetics (2005). p. 35–49.

[16] Wang Z Deurenberg P Wang W Pietrobelli A Baumgartner RN Heymsfield SB. Hydration of fat-free body mass: new physiological modeling approach. Am J Phys. (1999a) 276:E995–E1003. doi: 10.1152/ajpendo.1999.276.6.E995, PMID: 10362610

[17] Lohman T. Assessment of body composition in children. Pediatr Exerc Sci. (1989) 1:19–30. doi: 10.1123/pes.1.1.19 36696627

[18] Santos DA Matias CN Rocha PM Minderico CS Allison DB Sardinha LB. Association of basketball season with body composition in elite junior players. J Sports Med Phys Fitness. (2014) 54:162–73. PMID: 24509987

[19] Fuller NJ Jebb SA Laskey MA Coward WA Elia M. Four-component model for the assessment of body composition in humans: comparison with alternative methods, and evaluation of the density and hydration of fat-free mass. Clin Sci. (1992) 82:687–93. doi: 10.1042/cs0820687, PMID: 1320550

[20] Tanner JM. Growth at adolescence; with a general consideration of the effects of hereditary and environmental factors upon growth and maturation from birth to maturity. 2nd ed. Oxford: Blackwell Scientific Publications (1962).

[21] Ross WD Marfell-Jones MJ. Kinanthropometry. In: JD MacDougall HA Wenger HJ Green, editors. Phsysiological testing of the high-performance athlete. Champaign, IL: Human Kinetics Publishers (1991). p. 224–305.

[22] Du Bois D Bois D. A formula to estimate the approximate surface area if height and weight be known. Nutrition. (1989) 5:303. 2520314

[23] Silva AM Santos DA Matias CN Minderico CS Schoeller DA Sardinha LB. Total energy expenditure assessment in elite junior basketball players: a validation study using doubly labeled water. J Strength Cond Res. (2013) 27:1920–7. doi: 10.1519/JSC.0b013e31827361eb, PMID: 22990574

[24] Prosser SJ Scrimgeour CM. High-precision determination of 2H/1H in H2 and H2O by continuous-flow isotope ratio mass spectrometry. Anal Chem. (1995) 67:1992–7. doi: 10.1021/ac00109a014

[25] Wang Z Shen W Whithers RT Heymsfield SB. Multicomponent molecular level models of body composition analysis. In: SB Heymsfield TG Lohman Z Wang S Going, editors. The human body composition. Champaign, IL: Human Kinetics (2005). p. 163–176.

[26] Wang Z Deurenberg P Wang W Pietrobelli A Baumgartner RN Heymsfield SB. Hydration of fat-free body mass: review and critique of a classic body-composition constant. Am J Clin Nutr. (1999b) 69:833–41. doi: 10.1093/ajcn/69.5.833, PMID: 10232621

[27] Bland JM Altman DG Warner DS. Agreed statistics: measurement method comparison. Anesthesiology. (2012) 116:182–5. doi: 10.1097/ALN.0b013e31823d7784 22129533

[28] Silva AM Minderico CS Teixeira PJ Pietrobelli A Sardinha LB. Body fat measurement in adolescent athletes: multicompartment molecular model comparison. Eur J Clin Nutr. (2006) 60:955–64. doi: 10.1038/sj.ejcn.1602405, PMID: 16523205

[29] Wells JCK Williams JE Chomtho S Darch T Grijalva-Eternod C Kennedy K. Pediatric reference data for lean tissue properties: density and hydration from age 5 to 20 y. Am J Clin Nutr. (2010) 91:610–8. doi: 10.3945/ajcn.2009.28428, PMID: 20089731

[30] Borovnicar DJ Stroud DB Wahlqvist ML Strauss BJ. A neutron activation analysis facility for in vivo measurement of nitrogen and chlorine in children. Australas Phys Eng Sci Med. (1996) 19:252–63. PMID: 9060212

[31] Jebb SA Goldberg GR Jennings G Elia M. Dual-energy X-ray absorptiometry measurements of body composition: effects of depth and tissue thickness, including comparisons with direct analysis. Clin Sci. (1995) 88:319–24. doi: 10.1042/cs0880319, PMID: 7736701

[32] Laskey MA. Dual-energy X-ray absorptiometry and body composition. Nutrition. (1996) 12:45–51. doi: 10.1016/0899-9007(95)00017-8 8838836

[33] Roubenoff R Kehayias JJ Dawson-Hughes B Heymsfield SB. Use of dual-energy x-ray absorptiometry in body-composition studies: not yet a “gold standard.”. Am J Clin Nutr. (1993) 58:589–91. doi: 10.1093/ajcn/58.5.589 8237861

[34] Forbes GB. Human body composition: Growth, aging, nutrition, and activity. New York: Springer-Verlag (1987).

[35] Lyons-Reid J Kenealy T Albert BB Ward KA Harvey N Godfrey KM. Cross-calibration of two dual-energy X-ray absorptiometry devices for the measurement of body composition in young children. Sci Rep. (2022) 12:13862. doi: 10.1038/s41598-022-17711-0, PMID: 35974044 PMC9381538

[36] Park SS Lim S Kim H Kim KM. Comparison of two DXA systems, Hologic horizon W and GE lunar prodigy, for assessing body composition in healthy Korean adults. Endocrinol Metab. (2021) 36:1219–31. doi: 10.3803/EnM.2021.1274, PMID: 34911173 PMC8743584

[37] Francisco R Jesus F Nunes CL Cioffi I Alvim M Mendonca GV. Athletes with different habitual fluid intakes differ in hydration status but not in body water compartments. Scand J Med Sci Sports. (2023) 33:1072–8. doi: 10.1111/sms.14355 36951582

[38] De Faria ER Franceschini S Peluzio MCG Sant’Ana LFR Priore SE. Methodological and ethical aspects of the sexual maturation assessment in adolescents. Rev Paul Pediatr. (2013) 31:398–405. doi: 10.1590/S0103-05822013000300019, PMID: 24142325 PMC4182982

